# Trait and phylogenetic diversity provide insights into community assembly of reef‐associated shrimps (Palaemonidae) at different spatial scales across the Chagos Archipelago

**DOI:** 10.1002/ece3.3969

**Published:** 2018-03-26

**Authors:** Catherine E. I. Head, Heather Koldewey, Sandrine Pavoine, Morgan S. Pratchett, Alex D. Rogers, Michelle L. Taylor, Michael B. Bonsall

**Affiliations:** ^1^ Department of Zoology University of Oxford Oxford UK; ^2^ Conservation Programmes Zoological Society of London London UK; ^3^ Linacre College Oxford UK; ^4^ Centre for Ecology & Conservation University of Exeter Cornwall Campus Cornwall UK; ^5^ Centre d'Ecologie et des Sciences de la Conservation (CESCO UMR7204) Sorbonne Universités, MNHN, CNRS, UPMC, CP51 Paris France; ^6^ ARC Centre of Excellence for Coral Reef Studies James Cook University Townsville QLD Australia; ^7^ St Peter's College Oxford UK

**Keywords:** Chagos Archipelago, convergent and conserved evolution, coral reefs, cryptofauna, environmental filtering, metacommunity, phylogenetic diversity, trait diversity

## Abstract

Coral reefs are the most biodiverse marine ecosystem and one of the most threatened by global climate change impacts. The vast majority of diversity on reefs is comprised of small invertebrates that live within the reef structure, termed the cryptofauna. This component of biodiversity is hugely understudied, and many species remain undescribed. This study represents a rare analysis of assembly processes structuring a distinct group of cryptofauna, the Palaemonidae, in the Chagos Archipelago, a reef ecosystem under minimal direct human impacts in the central Indian Ocean. The Palaemonidae are a diverse group of Caridae (infraorder of shrimps) that inhabit many different niches on coral reefs and are of particular interest because of their varied habitat associations. Phylogenetic and trait diversity and phylogenetic signal were used to infer likely drivers of community structure. The mechanisms driving palaemonid community assembly and maintenance in the Chagos Archipelago showed distinct spatial patterns. At local scales, among coral colonies and among reefs fringing individual atolls, significant trait, and phylogenetic clustering patterns suggest environmental filtering may be a dominant ecological process driving Palaemonidae community structure, although local competition through equalizing mechanisms may also play a role in shaping the local community structure. Importantly, we also tested the robustness of phylogenetic diversity to changes in evolutionary information as multi‐gene phylogenies are resource intensive and for large families, such as the Palaemonidae, are often incomplete. These tests demonstrated a very modest impact on phylogenetic community structure, with only one of the four genes (PEPCK gene) in the phylogeny affecting phylogenetic diversity patterns, which provides useful information for future studies on large families with incomplete phylogenies. These findings contribute to our limited knowledge of this component of biodiversity in a marine locality as close to undisturbed by humans as can be found. It also provides a rare evaluation of phylogenetic diversity methods.

## 1 INTRODUCTION

Many processes are involved in determining how species coexist and assemble into communities. The niche‐based model of community assembly recognizes environmental filtering and limiting similarity as two important deterministic mechanisms responsible for structuring and maintaining communities (Webb, Ackerly, McPeek, & Donoghue, 2002). Environmental filtering is the process by which abiotic conditions favor species with certain adaptive traits necessary for survival in that environment (Webb et al., 2002). Limiting similarity refers to biotic interactions such as competition, mutualism, and facilitation, which tend to limit niche overlap and similar species coexisting leading to competitive exclusion (MacArthur & Levins, 1967). These processes act through density‐dependent mechanisms (limiting similarity) and density‐independent mechanisms (filtering) (Chase & Leibold, 2003; Chesson, 2000; Clark, 2009). This is supported by several studies of trait and phylogenetic diversity that indicate that communities are structured by ecological processes such as competition and environmental filtering (e.g., Best, Caulk, & Stachowicz, 2013; Cavender‐Bares, Keen, & Miles, 2006; Ingram & Shurin, 2009; Kraft & Ackerly, 2010; Mayfield, Boni, Daily, & Ackerly, 2005; Pavoine et al., 2014). An alternative model to explain community assembly, the “neutral model,” suggests that communities are shaped by stochastic processes operating independently upon individual species, which combine with random speciation and extinction to determine the composition of communities at local to regional scales (Hubbell, 2001). The neutral model is “a special case” of the niche model that assumes density dependence and that all species are equally fit (Adler, Hillerislambers, & Levine, 2007; Clark, 2009). Munoz and Huneman (2016) reviewed ecological equivalence and suggested that, although central to neutral theory, ecological equivalence can emerge at local and regional scales from niche‐based processes through equalizing and stabilizing mechanisms.

Patterns in trait and phylogenetic diversity may contribute to understanding ecological and evolutionary processes operating to shape specific species assemablages (Cadotte & Tucker, 2017; Mayfield & Levine, 2010; Pavoine & Bonsall, 2011; Webb et al., 2002). For instance, when comparing plots of different environments, clustering of both trait diversity and phylogenetic diversity can suggest that environmental filtering is the driving mechanism behind community assembly, and that the trait has phylogenetic signal (Pavoine, Baguette, & Bonsall, 2010; Table S1). Incorporating phylogenetic information can demonstrate how evolutionary history has shaped ecological processes and helps untangle the mechanisms behind community assembly (Pavoine & Bonsall, 2011; Webb et al., 2002). However, it is necessary to understand how traits evolve and change in order to interpret phylogenetic over‐dispersion versus clustering patterns as these patterns can occur through different mechanisms (Pavoine & Bonsall, 2011). Most notably phylogenetic over‐dispersion within a community can be a result of competition associated with traits that are conserved through evolutionary time, or environmental filtering processes associated with traits that have converged through evolution (Cavender‐Bares, Ackerly, Baum, & Bazzan, 2004; Kraft & Ackerly, 2010; Losos, 2008). However, taking phylogenetic and trait diversity together can distinguish between these mechanisms (Mayfield & Levine, 2010; Pavoine & Bonsall, 2011).

The relative influence of environmental filtering versus limiting similarity may depend on the habitat, spatial (Kraft & Ackerly, 2010; Swenson & Enquist, 2009), temporal (Pavoine, Vela, Gachet, de Bélair, & Bonsall, 2011), and taxonomic or phylogenetic scales (Cavender‐Bares et al., 2006). At biogeographical (continental or oceanic) scales, phylogenetic and/or trait clustering reflects biogeographic processes, such as currents and climatic factors (Webb et al., 2002). A regional scale or metacommunity (set of local communities linked by dispersal) can be divided into a local diversity component and a component associated with the difference between local communities (Pavoine & Bonsall, 2011; Swenson & Enquist, 2009; Veech, Summerville, Crist, & Gering, 2002).

Palaemonid shrimps (Family Palaemonidae, Infraorder: Caridae, Order: Decapoda) are highly diverse (De Grave & Fransen, 2011; De Grave, Fransen, & Page, 2015) and inhabit all oceans except the Arctic and Antarctic regions. They exhibit greatest diversity on Indo‐Pacific coral reefs (De Grave, 2001). An interesting characteristic of the Palaemonidae is their diverse lifestyles, for example, free‐living, semi‐symbiotic, and symbiotic. Symbiotic species form close associations with a range of hosts including molluscs, echinoderms, hard corals (Scleractinia), tunicates (ascidians), anemones (Actiniaria), and sponges (Porifera) (Bruce, 1977). Free‐living palaemonids have the general palaemonid body structure including well‐developed dentate rostrum and long slender chelae and pereiopods (Bauer, 2004). Palaemonid species with symbiotic associations with a host have evolved morphological adaptations in body shape, rostrum, mouthparts, eye‐design, and ambulatory legs (Bauer, 2004; Dobson, De Grave, & Johnson, 2014; Kou, Li, Chan, & Chu, 2014; Kou et al., 2013). For instance, *Coralliocaris* and *Jocaste* spp., which are considered live obligate coral‐dwellers (Head et al., 2015), have modified walking legs with a special appendage, called a dactyl, to improve their grip on their coral hosts (Bruce, 1977; Patton, 1994).

**Figure 1:**
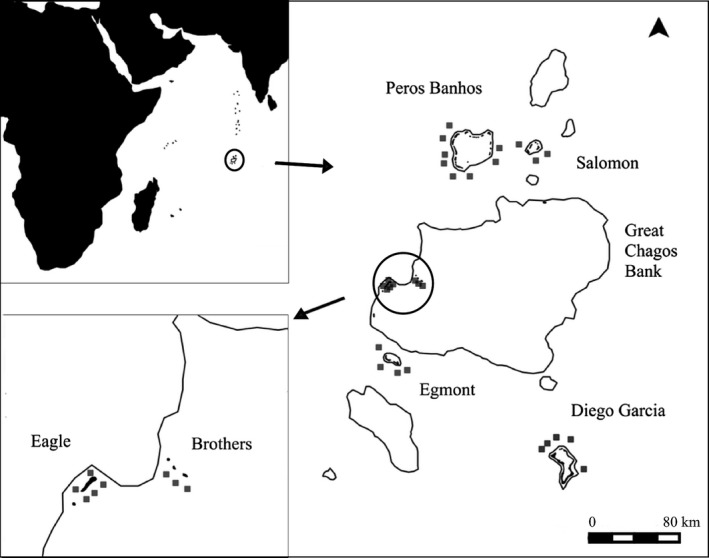
The Chagos Archipelago; gray squares represent the 28 sites where dead coral colonies were collected on the 2012 and 2013 expeditions. All outlines represent submerged atolls, with land represented by shading within the outlines. A close‐up of Eagle and Brothers Islands (part of the Great Chagos Bank) in the bottom left corner shows the distribution of the eight sites around these two islands

In this study, we investigate processes underlying community assembly of palaemonid shrimps on dead branching corals, across the Chagos Archipelago (British Indian Ocean Territory) in the central Indian Ocean (Figure 1). Specifically, we consider the relative influence of ecological and evolutionary processes in structuring palaemonid assemablages, using functional traits and phylogenetic information. We ask, *are the metacommunities and local communities different in terms of trait and phylogenetic diversity?* Clustering of trait values within a community can indicate environmental filtering at these spatial scale; however, competition through a reduction in fitness differences could also give rise to such a clustering pattern (Mayfield & Levine, 2010). Conversely if the traits show an over‐dispersed pattern then limiting similarity is most likely operating (Table S1). The phylogenetic pattern may also be clustered, over‐dispersed, or randomly distributed, at each spatial scale depending on the evolutionary conserved or convergent nature of the traits (Table S1). We evaluate whether different descriptions of evolution (the use of different genes) affect phylogenetic diversity patterns at each scale. Finally, we investigate whether there is phylogenetic signal in trait states and if this differs at different spatial scales. Together these analyses allow us to combine several indices of biodiversity: species abundance, trait diversity, phylogenetic diversity, and correlation between traits and phylogeny to begin to understand the processes underpinning Palaemonidae community structure.

## 2 MATERIALS AND METHODS

### 2.1 Sampling design

Sampling of dead branching corals (*n* = 65) was undertaken during two separate expeditions; from March to April 2012 and 2013 in the Chagos Archipeligo (Head et al., 2015). Sampling was conducted at 28 sites located on the outer reef and separated by at least 250 m across four atolls; Diego Garcia Atoll, Peros Banhos Atoll, Salomon Atoll, Eagle and Brothers Islands of the Great Chagos Bank, and Egmont Atoll (Figure 1). Between two to four dead *Acropora* or *Pocillopora* coral colonies of approximately 20 cm in diameter were sampled from 8 to 10 m depth at each site. To quantify cryptofaunal diversity, including all palaemonid shrimps, all macroorganisms inhabiting each coral head were carefully removed (Head et al., 2015). Sampling of cryptofauna on the coral colonies although extensive did not capture total estimated species richness as rarefaction curves were yet to plateau (Figure S1). Coral colonies were defined as being dead if they had no observable live polyps, evidence of turf and crustose coralline algae, and sometimes erosion. Palaemonid shrimp were identified to species and rare palaemonid species were catalogued in the Oxford University Museum of Natural History collections.

The sampling design allows measurement of beta diversity at three distinct spatial scales (1) among atolls, (2) among reefs within atolls, and (3) among coral colonies within reefs, to determine whether there was spatial structure to the community. We refer to the Archipelago as a meta‐population of palaemonids because biogeographic patterns of species distributions, prevailing currents, and modeling studies of ocean currents within the Archipelago all suggest good connectivity through‐out the Archipelago and some connectivity across the Indian Ocean (De Grave, 2001; Obura, 2012; Sheppard et al., 2012). In addition, studies on other taxa, such as crown‐of‐thorn‐starfish, provides evidence that the Archipelago acts as a stepping‐stone across the Indian Ocean (Sheppard et al., 2012). Furthermore, it can also be inferred from larval duration times of other marine shrimp species, for example, *Lysmata debelius (*family*: Hippolytidae*) with a larval duration of 63–158 days (Fletcher, Kotter, Wunsch, & Yasir, 1995) that distances over the three spatial scales of the sampling design should be well within the species’ dispersal range.

### 2.2 Phylogeny

**Figure 2:**
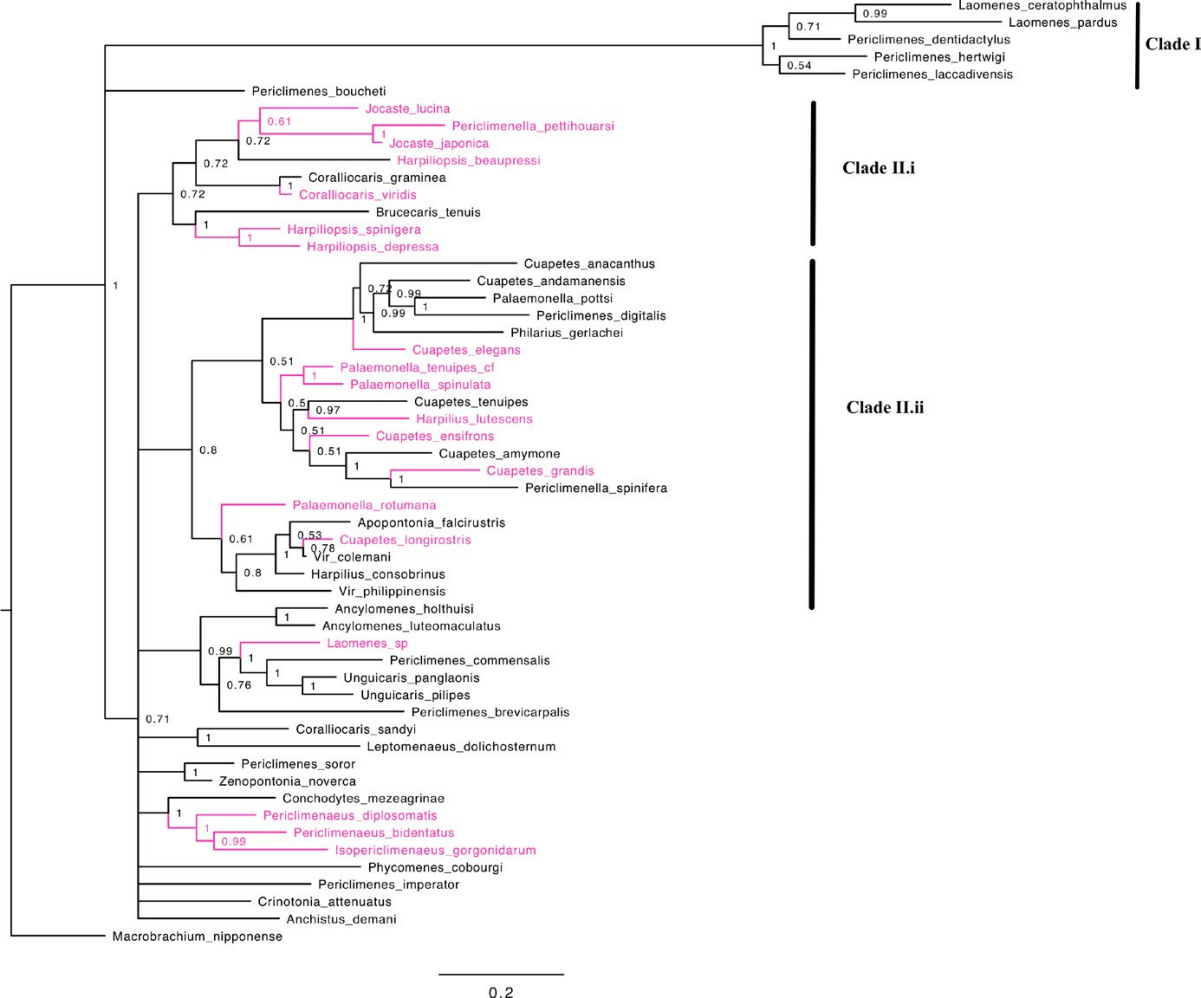
Bayesian inference phylogeny of 55 species from the family Palaemonidae, using a consensus sequence of four genes; 16S, Enolase, NaK, and PEPCK. Node support values represent Bayesian posterior probabilities. The species in magenta are those present in the Chagos metacommunity

Based on a previous Palaemonidae phylogenetic study (Kou et al., 2013), we used four genes to construct a focused community phylogeny; partial fragments of the 16S ribosomal RNA (rRNA) gene (~368 bp), and partial fragments of three nuclear genes; enolase (~405 bp), PEPCK (~521 bp), and NaK (~620 bp). Nineteen of the twenty species from the metacommunity were represented by at least two genes in the consensus phylogeny (see Table S2). Only *Exoclimenella maldevensis* was not included in the consensus phylogeny as we were only able to amplify the 16S gene for this species. As this species was rare in the community, occurring only once, it was excluded from further analysis. An additional 26 species were included in the phylogeny (from Chagos samples and available specimens on GenBank) to provide more information on the evolutionary relationships between species in the metacommunity. Phylogenetic trees were constructed under Bayesian Inference (BI) analysis in MrBayes v.3.2 (Ronquist et al., 2012) (see Table S3 for models of evolution used), on the online CIPRES Science Gateway (Miller & Schwartz, 2010) for the consensus alignment and for each gene tree separately. A composite metacommunity phylogeny was produced in APE using the phylogeny (Figure 2; Paradis, Claude, & Strimmer, 2004). All sequences were catalogued on GenBank. See Appendix S1 for a detailed methodology.

### 2.3 Traits

We measured the important and fundamental functional traits of body size and fecundity as measures of fitness. In addition, we gathered information from the literature on the species–host association, for example, hard coral, an important characteristic of the subfamily. As this community of palaemonids were collected from dead branching coral microhabitats, we split the host associations into four appropriate categories: hard coral (Scleractinia) associates, free‐living, semi‐symbiotic coral associates (palaemonid species which are generalists and have been recorded both free‐living and inhabiting corals), and sessile invertebrate associates. The last category refers to sessile invertebrates, such as sponges and tunicates, which are often found encrusting dead coral colonies. The palaemonid species we refer to here as “coral associates” are widely considered to be obligate live coral‐dwelling associates (Bruce, 1969, 1972, 1998) but we found large numbers of these species on dead coral colonies (Head et al., 2015) and consequently refer to them more generally as coral associates.

Carapace length was used as a proxy for body size and measured as the linear length of the carapace from the posterior of the orbital cavity to the most posterior tip of the carapace (Anger & Moreira, 1998). To measure fecundity, both mean egg size and total egg number were recorded. Twenty‐four gravid females were recorded from eight of the twenty species in the metacommunity. However, four gravid females were excluded from the analysis as a result of damage to the egg sac and/or suspected shedding of eggs during collection. The longest and shortest diameter of ten randomly selected eggs from each female were measured to the nearest 4 μm. Total egg number per female was also counted. To establish a measure of fecundity, linear regression and ANOVA were used to evaluate the relationship between egg number with egg size and with female body size. The relationships were plotted and a generalized additive model (gam) used to obtain a line of best‐fit. Egg number was then adjusted to take account of egg size as a measure of fecundity (see Appendix S2). Fecundity data were also interpolated, with respect to body size, for all species. The trait diversity analysis was then preformed on both the original fecundity data and the interpolated data to identify potential error caused by the small sample size of fecundity data (see Appendix S2).

### 2.4 Community phylogenetic analysis

To test for phylogenetic signal in the quantitative traits, we used the *K*‐statistic (Blomberg, Garland, & Ives, 2003), using the R package “phyltools” (Revell, 2012). This was preformed twice; once incorporating sampling error following Ives, Midford, and Garland (2007), as our data have within‐species variation which is not accounted for in other methods to the best of our knowledge, and for a second time without taking within species variation into account. Phylogenetic signal in the nominal trait of habitat association was tested using Maddison and Slatkin (1991) method which compares the minimum number of trait changes to the distribution of trait changes drawn from a null model. We used function “phylo.signal.disc” in R environment, developed by Enrico Rezende (Universidad Autònoma de Barcelona) (http://grokbase.com/k-for-discrete-unordered-traits). To investigate functional (trait) diversity and phylogenetic patterns in trait diversity across distinct spatial scales, we used the third proposition of Pavoine, Marcon, Ricotta, and Kembel (2016) to divide Rao's measure of diversity, named quadratic entropy (QE), (Rao, 1982) across spatial scale, using the R package “adiv” (Pavoine, 2017). This partitioning of diversity is adapted to unbalanced sampling design. Quadratic entropy is also relatively robust to sampling‐effects because this method is an extension of the Simpson index for functional and phylogenetic data, which gives high weight to common species (Lande, 1996). Therefore, rare species perhaps not identified by under‐sampling are unlikely to impact the index even if they are functionally or phylogenetically distinct from others. The QE index of diversity uses the phylogenetic tree, distributions of relative abundances of species in a community, and a matrix of trait distances among species obtained by Gower distance (Gower, 1971), to assess whether there is any phylogenetic and/or trait clustering in the metacommunities and local communities (Pavoine et al., 2010). Phylogenetic and/or trait clustering are measured using the beta diversity standardized effect size (SES), which calculates the observed beta diversity minus the mean of simulated beta diversities, divided by the standard deviation of simulated beta diversities. The trait‐based apportionment of quadratic entropy across spatial scales will be referred to as the trait quadratic entropy test (TQE) and that based on phylogenetic data as the phylogenetic quadratic entropy test (PQE). We measured beta diversity, using TQE and PQE, at three distinct spatial scales (1) among atolls, (2) among reefs within atolls, and (3) among coral colonies within reefs, to determine whether there was spatial structure to the community. To investigate how robust the community phylogenetic diversity patterns are to the evolutionary information used, we ran the apportionment of diversity (PQE test) on each gene tree separately in addition to the consensus phylogeny.

## 3 RESULTS

### 3.1 Are the metacommunities and local communities different in terms of trait and phylogenetic diversity?

When considering species abundances (see Figure S3 for illustration of species abundances), we detected both significant trait clustering in total trait diversity and phylogenetic clustering at the two local scales, that is, between reef sites within atolls (TQE beta Standardized Effect Size (*beta SES*) = 2.207, *p *=* *.012; *PQE beta SES *= 2.309, *p *=* *.014; both trait and phylogenetic diversity are lower locally than expected from the whole study area) and among coral colonies (*TQE beta SES *= 2.260, *p = *.013; *PQE beta SES = *4.646, *p = *.002). In contrast, there was no significant trait or phylogenetic diversity patterns detected at the highest spatial scale, that is among atolls within the archipelago, but the beta SES statistic showed negative values suggesting trait and phylogenetic diversity are over‐dispersed at this scale (Table 1). Interpolated fecundity data for all species did not affect the results of the total trait values (Appendix S3 and Table S4). When total trait diversity is decomposed into the three traits: body size, habitat association, and fecundity, we detect the same trait diversity patterns and significance values in habitat association across the spatial scales as for total trait diversity (Table 1a). However, although body size demonstrates the same trait diversity patterns across the spatial scales as total trait diversity these are not significant (Table 1a). Fecundity could not be tested separately due to the small number of individuals found to be fecund but interpolated fecundity data showed the same trait diversity patterns as body size (Table S4).

**Table 1:** Results of the partitioning of quadratic entropy at three spatial scales, using species abundance, and (a) traits, (b) phylogeny. Coral colonies within sites, sites within atolls, and atolls were evenly weighted. Beta SES = standardized effect size (observed beta diversity − mean of simulated beta diversities)/standard deviation of simulated beta diversities. *p‐value lower than .05. If beta SES values are negative community structure is over‐dispersed, if positive the community structure is clustered

	Beta diversity					
	Among atolls		Among sites within atolls		Among coral colonies within sites	
	SES	*p*‐Value	SES	*p*‐Value	SES	*p*‐Value
(a) Trait						
Total trait diversity	−1.003	.283	2.207	.012*	2.260	.013*
Body size	0.025	.985	1.073	.332	1.379	.159
Habitat association	−1.122	.239	2.402	.011*	2.603	.014*
(b) Phylogeny						
Consensus	−1.291	.156	2.309	.014*	4.646	.002*
16S gene	−1.279	.171	2.412	.014*	4.649	.002*
Enolase gene	−1.590	.091	2.468	.013*	4.875	.002*
NaK gene	−8.615	.375	2.187	.027*	3.667	.004*
PEPCK gene	−1.146	.196	1.653	.112	3.506	.004*

### 3.2 Does the evolutionary information (gene trees) affect the phylogenetic diversity patterns of metacommunities and local communities?

The same pattern in phylogenetic composition of the metacommunities and local communities is detected across the four different gene trees as for the consensus tree with the exception of phylogenetic diversity among reef localities within atolls using only PEPCK gene tree which although still demonstrating clustering is not significant (Table 1b).

### 3.3 Is there phylogenetic signal in trait states? And are the phylogenetic signals observed on the overall metacommunity different from the phylogenetic signals observed within local communities?

At the metacommunity scale, body size has a weaker phylogenetic signal (i.e., closely related species are less similar in body size) than would be expected under a Brownian motion model of evolution, when accounting for within‐species variation and the phylogenetic signal was not significant (*K *=* *0.47, σ = 3.58, *p = *.716). Fecundity had a stronger phylogenetic signal although still nonsignificant (*K *=* *0.91, *p = *.109), but the among‐species variation, once within‐species variation had been controlled for, was high (σ  =  62.23). When not accounting for within‐species variation, the pattern of phylogenetic signal did not change significantly for either palaemonid fecundity (*K = *1, *p = *.501) or body size (*K = *0.56, *p = *.46). Host association showed no significant phylogenetic signal across the metacommunity nor at the local scale of reefs within atolls *(Maddison and Slatkin test, p = .99*). At a local scale (among reefs within atolls), phylogenetic signal for body sizes showed the same trend as at the metacommunity scale with body size at each atoll having a weaker phylogenetic signal than would be expected under a Brownian motion model of evolution, when accounting for within‐species variation (see Table 2 for *K* and σ statistics). Body size at Diego Garcia and Salomon had the strongest phylogenetic signals. Phylogenetic signal could not be tested for fecundity at the local scales because the number of gravid females per atoll was too small to produce meaningful results.

**Table 2:** Phylogenetic signal in body size per atoll using the Blomberg's *K* statistic. If *K* is less than 1, there is less phylogenetic signal than would be expected by chance under a Brownian model of evolution. σ shows the variation around the *K* statistic after controlling for intra‐specific variation

Atoll	Body size		
	*K* statistic	σ	*p*‐Value
Brothers	0.69	2.70	.698
Diego Garcia	0.92	0.80	.284
Eagle	0.82	0.56	.372
Egmont	0.85	0.44	.453
Peros Banhos	0.47	4.02	.827
Salomon	0.98	0.60	.063

## 4 DISCUSSION

The mechanisms driving palaemonid community assembly and maintenance in Chagos show distinct spatial patterns. Different processes are known to act at different spatial scales, and this has been demonstrated particularly clearly in forest ecosystems (Kraft & Ackerly, 2010; Ricklefs, 1987). In Chagos, the QE tests demonstrate spatial hierarchy with significant total trait and phylogenetic clustering at the local community scales, among reef localities fringing each atoll and among coral colonies. Both trait and phylogenetic clustering suggest that environmental filtering could be an important ecological process acting at the local community level (Table S1; Pavoine & Bonsall, 2011; Webb et al., 2002), although competition can also result in these clustering patterns (Cadotte & Tucker, 2017; Mayfield & Levine, 2010) (see below discussion). Within the metacommunities and local communities, weak phylogenetic signal in body size and a stronger phylogenetic signal in fecundity at the highest spatial scale were detected but nonsignificant, indicating these traits are at least partially regulated by the phylogeny through both trait conservatism and convergence. Pavoine, Gasc, Bonsall, and Mason (2013) showed that phylogenetic signal in traits does not always imply similarities in functional and phylogenetic diversity patterns. Here we show that, inversely, similarities in functional and phylogenetic patterns do not always imply phylogenetic signal in traits. Phylogenetic patterns were also largely robust to changes in evolutionary information as discussed below.

### 4.1 Trait and phylogenetic clustering

Environmental filtering results in the evolutionary selection of species with a similar set of traits adapted to the specific environmental conditions (Webb et al., 2002). Trait and phylogenetic clustering suggest that environmental filtering could be an important mechanism acting on the traits of fecundity and habitat association within local communities in the Chagos Archipelago, and therefore, potentially underpinning palaemonid species distribution to some extent in these local communities. However, these observed patterns can also reflect the effects of competition, or the combined effects of both the environment and local competition, and it is hard to completely disentangle these effects (Cadotte & Tucker, 2017). Nonetheless correlations between palaemonid community structure and specific environmental variables in the Chagos Archipelago support the inference that environmental filtering is an important mechanism acting on palaemonid local communities (Head, 2015). In multiple geographical locations, it has been well reported that coral associate abundance, species richness, and biomass increase with coral colony size, and complexity in live coral colonies (Abele, 1976; Abele & Patton, 1976; Coles, 1980; Vytopil & Willis, 2001) and to a lesser extent in dead coral colonies (Enochs & Manzello, 2012; Enochs, Toth, Brandtneris, Afflerbach, & Manzello, 2011). While our previous work in the Chagos Archipelago indicates that size and complexity of habitable space of the dead coral colony host is likely an environmental filter that acts on palaemonid body size (Head et al., 2015). However, even though palaemonid body size demonstrated a clustered pattern, perhaps inferring environmental filtering is acting on the community, it was not significant. Whereas significant phylogenetic and trait clustering of palaemonid habitat association at the most local community scales suggests that environmental filtering may be at least partly responsible for determining the abundance and community structure of free‐living, hard coral associates, hard coral semi‐symbionts, and sessile invertebrate host associates. It is perhaps surprising that no clustering in habitat association was detected at the metacommunity scale because *J. lucina* and *H. spinigera*, the two most dominant species (Figure S3), both have the same habitat association (they are associated with hard corals).

In contrast to the local community scales, phylogenetic and trait diversity were mainly over‐dispersed (with the exception of body size), although the patterns were nonsignificant, at the highest spatial scale; among atolls, suggesting that other processes such as competition and facilitation may be more important at this scale. In addition, the lack of environmental filtering could result from heterogeneous environmental conditions across the archipelago (Sheppard et al., 2012) and/or because coral reefs are highly complex ecosystems with many environmental drivers that are often hard to tease apart (Bauman, Feary, Heron, Pratchett, & Burt, 2013; Graham & Nash, 2012; Hughes et al., 2017).

### 4.2 Evolutionary information

PQE measures phylogenetic diversity using a matrix of genetic pairwise distances consisting of the proportion of nucleotides at which each two sequences differ (Nei & Kumar, 2000). Palaemonid phylogenetic diversity patterns were relatively robust to the use of different genetic information, that is, 16S, Enolase, NaK, and PEPCK genes. The change in genetic information had an effect in a single case; that is, the clustering in phylogenetic diversity was no longer significant when considering only PEPCK gene sequences at the local community scale of reef localities fringing atolls. This maybe because the PEPCK gene had less original data than the other genes, as we were unable to amplify PEPCK sequences for *Periclimenaus pettihouarsi*,* Palaemonella spinulata,* and *Periclimenaeus bidentatus,* rather than the proportion of nucleotide differences. All three species were rare in the community and well dispersed throughout the multi‐gene phylogeny (Figure 2). Boyle and Adamowicz (2015) also found that estimates of phylogenetic community structure from distance matrices derived from gene trees were generally concordant with those generated from multi‐gene trees using net relatedness index (NRI) and nearest taxon index (NTI). The relatively robust patterns in palaemonid phylogenetic diversity give us high confidence in our results, and this is particularly important for the Palaemonidae because they are a large family whose phylogeny is incomplete (De Grave et al., 2015; Gan, Li, Chan, Chu, & Kou, 2015).

### 4.3 Phylogenetic signal at all spatial scales

Recent developments in the palaemonid phylogeny suggest that varied host associations have developed through convergent evolution with species independently invading their hosts (Gan et al., 2015; Kou et al., 2013) as well as through host switching (Horka, de Grave, Fransen, Petrusek, & Duris, 2016). In Chagos, we found a lack of phylogenetic signal in habitat association suggesting palaemonid habitat associations have evolved independently of phylogeny and that close relatives are not more similar than distant relatives (Blomberg et al., 2003). Analysis on body size revealed that this trait had no significant phylogenetic signal in local communities within Chagos and across the metacommunity. Despite the stronger phylogenetic signal for fecundity, there was also high variability around this signal. So, while fecundity was regulated by phylogeny and environment (the latter only at the two lower spatial scales), trait convergence within the phylogeny was considerable. The weak or partial phylogenetic conservatism could be a result of evolutionary lability in traits, where some lineages experience higher rates of trait evolution than others (Ackerly, 2009; Blomberg et al., 2003; Pavoine et al., 2014).

## 5 CONCLUSIONS

Here we have investigated a community phylogenetic approach for studying marine systems. This study represents a rare investigation into the community assembly processes that structure a marine invertebrate community. The mechanisms driving palaemonid community assembly and maintenance in Chagos show distinct spatial patterns. Both environmental filtering and the phylogeny likely influence trait diversity and patterns of coexistence to some extent within Palaemonidae shrimps occupying individual coral colonies and among reef localities fringing each atoll in the archipelago. The choice of input gene tree had modest impact on the phylogenetic community structure, which is useful information for future studies as construction of multi‐gene phylogenies is a resource intense process; however, these results may also be taxon specific. Furthermore, phylogenetic signal was weak and not significant (body size, host association) or highly variable (fecundity), both within local communities and at the metacommunity level, suggesting trait convergence and lability of trait evolution could be key processes determining species distribution. Evidence of trait convergence means evolutionary history should be used in conjunction with life‐history traits to understand the patterns and processes underpinning community composition, as has recently been advocated by others (Kraft & Ackerly, 2010; Pavoine et al., 2014). As relatively little is known about the life‐history strategies of palaemonid species (Dobson et al., 2014; Horka et al., 2016; Kou et al., 2014), we chose to focus on the fundamental traits of body size and fecundity and also habitat association. However, as our knowledge increases it will be important to identify other key functional traits associated with the family, to improve our understanding of community assembly. Overall in this study, clustering patterns suggest that environmental filtering could be a significant ecological process acting at the local community scales, among reef sites within atolls and among coral colonies, and evolutionary mechanisms (trait convergence, labile rates of trait diversification) driving compositional patterns in palaemonid local communities in the Chagos Archipelago and possibly across the archipelago metacommunity.

## CONFLICT OF INTEREST

None declared.

## AUTHOR CONTRIBUTIONS

MB, CH, and AR conceived the ideas and designed methodology; CH, HK, and MP collected the data; CH, SP, and MT analyzed the data; CH led the writing of the manuscript. All authors contributed critically to drafts and gave final approval for publication.
