# Supplementary material for: Trait and phylogenetic diversity provide insights into community assembly of reef‐associated shrimps (Palaemonidae) at different spatial scales across the Chagos Archipelago
Source: Ecol Evol. 2018 Mar 26;8(8):4098–107. doi: 10.1002/ece3.3969 (PMC5916300; doi:10.1002/ece3.3969)
Supplement: Supplementary file 1 [file ECE3-8-4098-s001.docx]

**Supporting Information**

**Appendix S1**

**Materials & Methods**

**Figure S1** Rarefraction curves for the infra-order Caridea, to which the Palaemonidae belongs, for the Archipelago and for each atoll.

*Phylogeny*

The pleopods, eggs or abdominal tissue were used for DNA extraction using Qiagen’s DNeasy Blood and Tissue kits resulting in 100µl elutions. Four genes were targeted in accordance with a previous Palaemonidae phylogenetic study (Kou et al 2013); partial fragments of the 16S ribosomal RNA (rRNA) gene (~368bp), and partial fragments of three nuclear genes enolase (~405bp), PEPCK (~521bp), and NaK (~620bp). Polymerase chain reaction (PCR) amplifications were carried out in 18μl volume reactions using; 12µl Master Mix HotStarTaq (Qiagen), 3µl DNA template, 0.6µl RNase-free water (Qiagen), and 2.4µl (4µM) primer mix (2µl forward, 2µl reverse, and 96µl RNase-free water). For the 16S gene and enolase genes the forward and reverse primers 16S-l2/1472 (Schubart et al., 2002) and EA2/ES2 (Tsang et al., 2011) amplified the respective gene consistently. However, amplification of PEPCK and NaK genes required testing of different primer set combinations for each specimen. The successful forward and reverse primer combinations were either For2/Rev or For/Rev3 for PEPCK and For-b/Rev or For-b/Rev2 for NaK (Tsang et al., 2008, Kou et al., 2013).  PCR thermal cycle conditions for the enolase, NaK and PEPCK genes were; initial denaturation of 15min at 94^o^C, followed by 40 cycles of denaturisation at 94^o^C for 30s, annealing at 55^o^C/56.5^o^C/56^o^C accordingly for 40s, extension at 72^o^C for 1 min, and a final extension at 72^o^C for 10mins. Thermal cycle parameters for the 16S was slightly different after the initial 15 min at 95^o^C; with 40 cycles of 60s at 94^o^C, 110s at annealing temperature of 46^o^C, and 110s at 72^o^C; followed by 10 min at 72^o^C. PCR reactions were checked using gel electrophoresis, and successful amplifications were purified using ExoSap-IT (Affymetrix) following the manufacturer's instructions.  Automated sequencing was performed on purified products in both directions with the aforementioned primers using an Applied Biosystems 3730xl DNA Analyzer.

Sequences were paired and edited using chromatograph visualisations in Geneious 6.1.5 (Biomatters Ltd., Auckland, New Zealand).  Each gene multi-alignment was compiled using the Geneious alignment function using default settings (Biomatters Ltd.), and then checked and edited.  Some regions of the 16S gene appeared highly variable and were difficult to align with confidence. So the 16S alignment was run through the GBlocks server using the less stringent option which allows for less strict flanking positions (Castresana, 2000), in order to eliminate the most highly divergent regions using a reproducible set of conditions. Consequently 133 nucleotides (nt) were eliminated reducing the partial 16S rRNA fragment to 368nt. We retained 55 species in the phylogenetic analysis for which we had reliable sequences for at least two genes (Table S1), as it has been demonstrated that utilising even incomplete data in phylogenies can be beneficial (Wiens, 2003, Wiens and Tui, 2012). These included 19 species from the Chagos metacommunity (Table S1). Sequences were catalogued on GenBank.

Phylogenetic trees were constructed for the consensus alignment (all four genes combined where available; Table S1), 16S and enolase combined, and for each gene separately.  To root the tree, each tree included an outgroup, *Macrobrachium nipponense,*a species belonging to the superfamily Palaemonoidea within which the family Palaemonidae resides (Kou et al. 2013).  To select appropriate models of molecular evolution on which accurate phylogenetic inference largely depends (Simon et al., 2006), partitioning was undertaken in PartitionFinder v.1.1 software (Lanfear et al., 2012).  PartitionFinder groups together sites in the alignment that are assumed to have been shaped by similar evolutionary processes, and then estimates independent substitution models for each group of sites (Lanfear et al. 2012). A 10-partition scheme obtained the highest support under Bayesian Information Criterion (BIC), detailed in Table S2.  Using these models of evolution (Table S2), phylogenies were constructed under Bayesian Inference (BI) analysis in MrBayes v.3.2 (Ronquist et al., 2012), on the on-line CIPRES Science Gateway (Miller and Schwartz, 2010). Metropolis-coupled Monte Carlo Markov Chains (MCMC) were run for 30 million generations (x8 chains, temp =0.05), with trees sampled every 3000 generations, resulting in 10 million trees. The parameters of nucleotide frequencies, substitution rates, gamma shape, and invariant-sites proportion were unlinked across partitions. A flat Dirichlet prior distribution allowed rates to vary, to account for rate variation among partitions (Marshall et al., 2006).  Tracer v.1.6 was used to ascertain if convergence had been obtained (Rambaut and Drummond, 2007). If standard deviation of partition frequencies was <0.01, potential scale reduction factor (PSRF) was ~1.00, effective sample sizes (ESS) were >200, and the shape of the stationary posterior-distribution trace was a ‘straight hairy caterpillar’ the data were considered to have converged (Drummond and Rambaut, 2007). Once convergence was reached a summary tree was constructed by discarding the first 10% of trees as burn-in, and then using a 50% majority rule consensus tree to construct the summary tree and estimate posterior probabilities (PP) in MrBayes.

**Table S1** Possible explanatory mechanisms for each phylogenetic and trait pattern combination (adapted from Pavoine et al. 2010). EF=Environmental filtering, LS=Limiting similarity.

| Phylogenetic pattern | Trait pattern | | |
| --- | --- | --- | --- |
|  | Clustering | Overdispersion | Randomness |
| Clustering | EF; phylogenetic signal; low α trait diversity | LS; convergence; high α trait diversity | Critical conserved traits involved in EF have been omitted; α random diversity |
| Overdispersion | EF; convergence; low α trait diversity | LS; phylogenetic signal; high α trait diversity | Critical conserved traits involved in LS have been omitted; random α trait diversity |
| Randomness | EF; labile traits; low α trait diversity | LS; labile traits; highα trait diversity | Neutral processes; balance between EF and LS |

**Table S2** Genes sequenced for the 55 species comprising the partial Palaemonidae phylogeny, including 19 species from the Chagos metacommunity.

| Genus | Species | Species in Chagos meta  community | GenBank accession number/ Sequence IDs | | | |
| --- | --- | --- | --- | --- | --- | --- |
|  |  |  | 16S | Enolase | NaK | PEPCK |
| Family: Palaemonidae | |  |  |  |  |  |
| *Anchistus* | *Anchistus demani* | No | CH0996 | CH0996 | CH0996 | CH0995 |
| *Ancylomenes* | *Ancylomenes holthuis* | No | JX025220 | JX537915 | JX467438 | JX435436 |
|  | *Ancylomenes luteomaculatus* | No | JX025222 | JX537916 | JX467439 | JX435437 |
| *Apopontonia* | *Apopontonia falcirustris* | No | MA0150b | MA150b | MA150b | MA150b |
| *Brucecaris* | *Brucecaris tenuis* | No | JX025218 | JX537917 | JX467440 | JX435438 |
| *Conchodtytes* | *Conchodtytes mezeagrinae* | No | CH0902 | CH0902 | CH0902 | CH0902 |
| *Coralliocaris* | *Coralliocaris graminea* | No | KF38361 | KF738298 | KF738343 | ------------ |
|  | *Coralliocaris sandyi* | No | KF38362 | KF738299 | KF738344 | ------------ |
|  | *Coralliocaris viridis* | Yes | CH0948 | CH0948 | CH0948 | CH0948 |
| *Crinotonia* | *Crinotonia attenuatu* | No | ----------- | JX537918 | JX467441 | JX435439 |
| *Cuapetes* | *Cuapetes amymone* | No | JX025216 | JX537919 | JX467442 | JX435440 |
|  | *Cuapetes anacanthus* | No | JX025215 | JX537920 | JX467443 | JX435441 |
|  | *Cuapetes andamanensis* | No | JX025214 | JX537921 | JX467444 | JX435442 |
|  | *Cuapetes elegans* | Yes | JX025213 | JX537922 | JX467445 | JX435443 |
|  | *Cuapetes ensifrons* | Yes | JX025212 | JX537923 | JX467446 | JX435444 |
|  | *Cuapetes grandis* | Yes | JX025211 | JX537924 | JX467447 | JX435445 |
|  | *Cuapetes longirostris* | Yes | CH1172 | CH1172 | CH1172 | CH1172 |
|  | *Cuapetes tenuipes* | No | JX025209 | JX537925 | JX467448 | JX435446 |
| *Harpiliopsis* | *Harpiliopsis beaupressi* | Yes | MA312b | MA312b | MA312b | JX435447 |
|  | *Harpiliopsis depressa* | Yes | CH0737 | CH0836 | CH0836 | CH0836 |
|  | *Harpiliopsis spinigera_* | Yes | CH0662 | CH0662 | CH0662 | CH0662 |
| *Harpilius* | *Harpilius consobrinus* | No | CH1019 | CH1019 | CH1019 | CH1019 |
|  | *Harpilius lutescens* | Yes | CH1009 | CH1009 | JX467450 | CH1009 |
| *Isopericlimenaeus* | *Isopericlimenaeus gorgonidarum* | Yes | CH0684 | ----------- | CH0684 | CH0684 |
| *Jocaste* | *Jocaste japonica* | Yes | CH0946 | CH0333 | CH0333 | CH0333 |
|  | *Jocaste lucina* | Yes | CH0128 | CH0737 | CH0737 | CH0128 |
| *Laomenes* | *Laomenes ceratophthalmus* | No | JX025203 | JX537928 | JX467451 | JX435449 |
|  | *Laomenes pardus* | No | JX025202 | JX537929 | JX467452 | JX435450 |
|  | *Laomenes sp* | Yes | CH0807 | CH0807 | CH0807 | CH0807 |
| *Leptomenaeus* | *Leptomenaeus dolichosternum* | No | JX025201 | JX537930 | JX467453 | JX435451 |
| *Palaemonella* | *Palaemonella pottsi* | No | JX025198 | JX537932 | JX467455 | JX435453 |
|  | *Palaemonella rotumana* | Yes | MA540 | MA540 | MA540 | MA056 |
|  | *Palaemonella spinulata cf* | Yes | CH0623 | KF738304 | KF738352 | ----------- |
|  | *Palaemonella tenuipes cf* | Yes | CH0923 | CH0923 | CH0923 | CH0923 |
| *Periclimenaeus* | *Periclimenaeus bidentatus* | Yes | KF738368 | CH0402 | KF738353 | ----------- |
|  | *Periclimenaeus diplosomatis* | Yes | CH0952b | CH0952 | CH1200 | CH0952 |
|  | *Periclimenaeus pettihouarsi* | Yes | CH1085 | CH1085 | ------------ | ----------- |
| *Periclimenella* | *Periclimenella spinifera* | No | JX025194 | JX537933 | JX467456 | JX435454 |
| *Periclimenes* | *Periclimenes boucheti* | No | JX025192 | JX537934 | JX467457 | JX435455 |
|  | *Periclimenes brevicarpalis* | No | JX025191 | JX537935 | JX467458 | JX435456 |
|  | *Periclimenes commensalis* | No | JX025190 | JX537936 | JX467459 | JX435457 |
|  | *Periclimenes dentidactylus* | No | JX025189 | JX537937 | JX467460 | JX435458 |
|  | *Periclimenes digitalis* | No | JX025188 | JX537938 | JX467461 | JX435459 |
|  | *Periclimenes hertwigi* | No | JX025186 | JX537939 | JX467462 | JX435460 |
|  | *Periclimenes imperator* | No | JX025185 | JX537940 | JX467463 | JX435461 |
|  | *Periclimenes laccadivensis* | No | JX025184 | JX537941 | JX467464 | JX435462 |
|  | *Periclimenes soror* | No | JX025178 | JX537942 | JX467465 | JX435463 |
| *Philarius* | *Philarius gerlachei* | No | JX025177 | JX537943 | JX467466 | JX435464 |
| *Phycomenes* | *Phycomenes cobourgi* | No | JX025174 | JX537944 | JX467467 | JX435465 |
| *Unguicaris* | *Unguicaris panglaonis* | No | JX025172 | JX537945 | JX467468 | JX435466 |
|  | *Unguicaris pilipes* | No | JX025171 | JX537946 | JX467469 | JX435467 |
| *Vir* | *Vir colemani* | No | CH0989 | CH0989 | CH0989 | CH0949 |
|  | *Vir philippinensis* | No | JX025170 | JX537947 | JX467470 | JX435468 |
| *Zenopontonia* | *Zenopontonia noverca* | No | CH1141 | CH1141 | ----------- | CH1141 |
| Superfamily: Palaemonoidea | |  |  |  |  |  |
| *Macrobrachium* | *Macrobrachiumnipponense* | No | JX435435 | JX537948 | JX467471 | JX435469 |

**Table S3**  Partition scheme and best-fit models of evolution used in Bayesian Inference (BI) analysis.

| Gene and codon position number | Partition delineation | Best-fit model of evolution |
| --- | --- | --- |
| Enolase 1st | 1-405\3 | K80+I+G |
| Enolase 2nd | 2-405\3 | K80+I+G |
| Enolase 3rd | 3-405\3 | GTR+G |
| NaK 1st | 406-1026\3 | GTR+I+G |
| NaK 2nd | 407-1026\3 | GTR+I+G |
| NaK 3rd | 408-1026\3 | GTR+G |
| PEPCK 1st | 1027-1548\3 | GTR+I+G |
| PEPCK 2nd | 1028-1548\3 | GTR+I+G |
| PEPCK 3rd | 1029-1548\3 | GTR+I+G |
| 16S | 1549-1992 | K80+I+G |

**Appendix S2**

**Results**

*Phylogeny*

The consensus phylogeny consists of two major clades, clade I is well supported (Bayesian posterior probability (PP)=1) and consists of only five species from the genera *Periclimenes* and *Laomenes*, species from both these genera are also placed in clade II. Clade II is moderately well supported (PP=0.7) but the basal relationships with its nested clades are not well resolved. All of the Chagos metacommunity species are dispersed throughout clade II but with clusters of seven species and eight species in two of the largest nested clades, clade II.i (PP=0.71) and II.ii (PP=0.81) respectively (Fig. S1). The seven species from the metacommunity, all clustered in clade II.i, are all scleractinian associates, with the exception of the free-living species *Periclimenella pettihouarsi*. The eight species dispersed throughout clade II.ii are all free living and sometimes associated with scleractinian corals, with the exception of *Harpilius lutescens,* which is purely a Scleractinia associate.  The four remaining species from the metacommunity not located in these two larger nested clades are all associates of sessile organisms encrusting on the branches of the dead corals; *Laomenes*spp. (crinoid associate), *Isopericlimenaeus gorgonidarum* (encrusting sponge associate), *Periclimenaeus bidentatus* (encrusting sponge associate) and *Periclimenaeus dactylodon* (encrusting ascidian associate). *I.gorgonidarum, P.bidentatus*and*P.dactylodon* are all clustered together in a small clade. Whilst *Laomenes spp.* appears to be a sister species of three other crinoid associates; *Perclimenes commensalis, Unguicaris panglaonis, Unguicaris pilipes*.

**Appendix S3**

*Traits*

Linear regression demonstrated that egg size was significantly negatively correlated with egg number (*t* = -2.65, *p* = 0.02), but neither egg number (*t* = 0.15, *p* = 0.88) nor egg number adjusted for egg size (*t* = 0.37, *p* = 0.72) had a significant linear relationship with female body size (Fig. S2). Therefore, egg number was adjusted to account for egg size only (egg number was multiplied by egg size), and used as a measure of fecundity.

In order to assess the impact of the small proportion of fecundity measures on the community phylogenetic analysis (specifically on the TQE) we also interpolated fecundity measures with respect to bodysize for all individuals using the GAM line of best-fit. The TQE analysis was then run on the original dataset (Table 1a) and also a second time on the interpolated fecundity data allowing comparison of results (Table S4).

**Figure S2** Scatterplots, with lines of fit best, demonstrating **(a)** the significant relationship between the number of eggs and average egg size, and **(b)** the non-significant relationship between number of eggs adjusted for egg size and female body size. Hence, egg number adjusted for egg size is used as a measure of fecundity.

**Table S4** Results of the partitioning of traits quadratic entropy at three spatial scales, using species abundance and the extrapolated fecundity measure. Coral colonies within sites, sites within atolls and atolls were evenly weighted. Beta SES=standardized effect size (observed beta diversity - mean of simulated beta diversities)/standard deviation of simulated beta diversities. **P*-value lower than 0.05. If beta SES values are negative community structure is over-dispersed, if positive the community structure is clustered.

|  | **Beta diversity using extrapolated fecundity measures:** | | | | | |
| --- | --- | --- | --- | --- | --- | --- |
|  | **among atolls** | | **among sites within atolls** | | **among coral colonies within sites** | |
| **Trait** | **SES** | ***P*-value** | **SES** | ***P*-value** | **SES** | ***P*-value** |
| **Total trait diversity** | -1.06 | 0.324 | 2.183 | 0.014* | 2.788 | 0.009* |
| **Fecundity** | -4.214 | 0.615 | 1.281 | 0.194 | 3.758 | 0.552 |


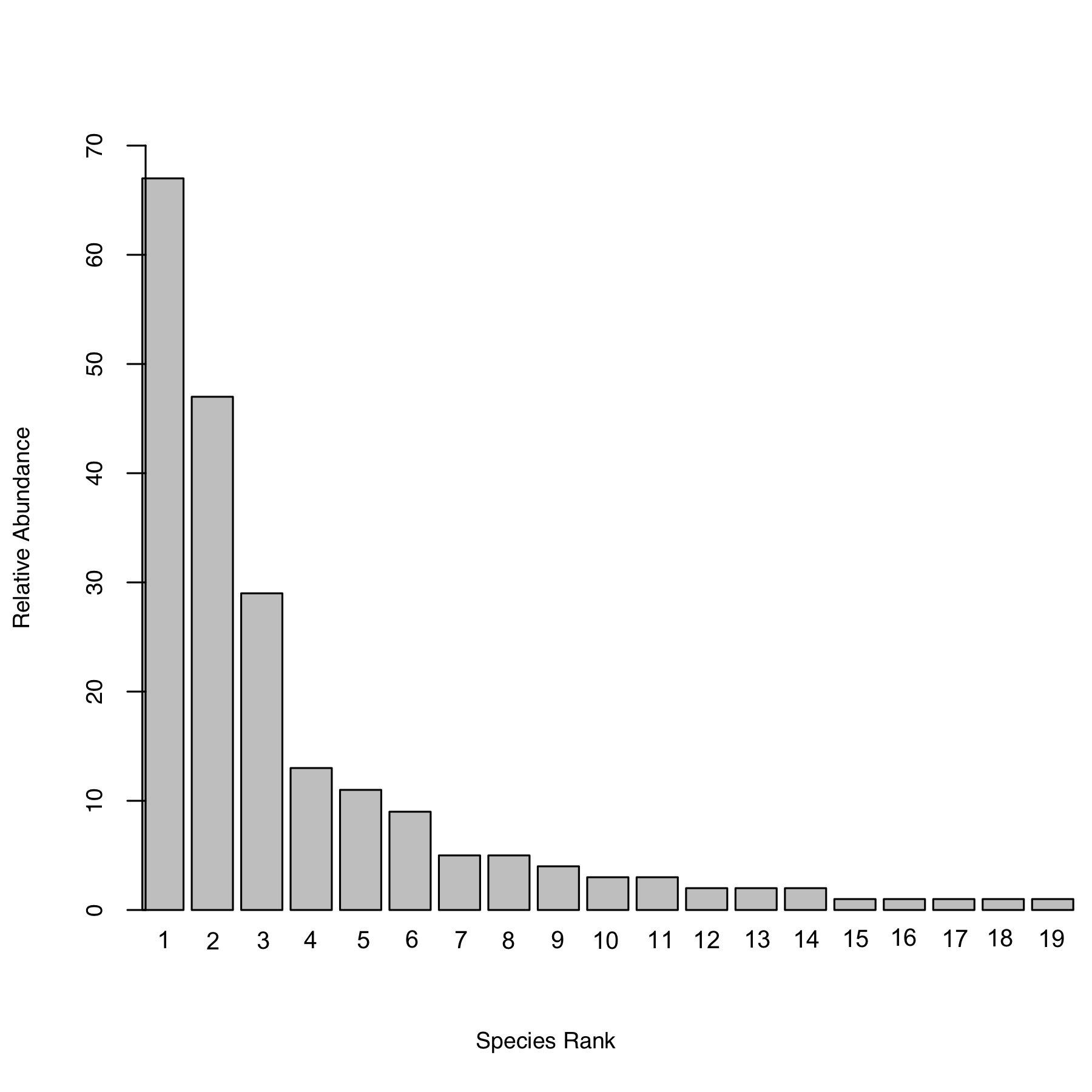


**Figure S3** Rank abundance plot of the palaemonids across the Chagos Archipelago. Ranks: *1=Jocaste lucina, 2=Harpiliopsis spinigera, 3=Cuapetes elegans, 4=Jocaste japonica, 5=Cuapetes grandis complex, 6=Palaemonella tenuipescf, 7=Harplius lutescens cf, 8=Harpiliopsis beaupressi, 9=Palaemonella rotumana cf, 10=Periclimenaeus bidentatus cf, 11=Harpiliopsis depressa, 12=Periclimenaeus diplosomatis cf, 13=Cuapetes longirostris cf, 14=Coralliocaris viridis, 15=Periclimenella pettithouarsi, 16=Palaemonella spinulata cf, 17=Laomenes sp., 18=Isopericlimenaeus gorgonidarum, 19=Cuapetes ensifrons.*

**References**

CASTRESANA, J. 2000. Selection of conserved blocks from multiple alignments for their use in phylogenetic analysis. . *Molecular biology and evolution,* 17**,** 540-552.

DRUMMOND, A. & RAMBAUT, A. 2007. BEAST: Bayesian evolutionary analysis by sampling trees. *BMC Evol. Biol.,* 7**,** 1-8.

KOU, Q., LI, X., CHAN, T. Y., CHU, K. H., HUANG, H. & GAN, Z. 2013. Phylogenetic relationships among genera of the Periclimenes complex (Crustacea: Decapoda: Pontoniinae) based on mitochondrial and nuclear DNA. *Molecular phylogenetics and evolution,* 68**,** 14-22.

LANFEAR, R., CALCOTT, B., HO, S. Y. & GUINDON, S. 2012. Partitionfinder: combined selection of partitioning schemes and substitution models for phylogenetic analyses. *Molecular biology and evolution,* 29**,** 1695-701.

MARSHALL, D. C., SIMON, C. & BUCKLEY, T. R. 2006. Accurate branch length estimation in partitioned bayesian analyses requires accommodation of among-partition rate variation and attention to branch length priors. *Syst. Biol.,* 55**,** 993–1003.

MILLER, M. A. & SCHWARTZ, T. 2010. Creating the CIPRES Science Gateway for inference of large phylogenetic trees. In: Proceedings of the Gateway Computing Environments Workshop (GCE),. *Institute of Electrical and Electronics Engineers (IEEE), Washington, District of Columbia***,** 1–8.

PAVOINE, S. & BONSALL, M. B. 2010. Measuring biodiversity to explain community assembly: a unified approach. *Biological reviews of the Cambridge Philosophical Society,* 86**,** 792-812.

RAMBAUT, A. & DRUMMOND, A. 2007. Tracer v1.5.

RONQUIST, F., TESLENKO, M., VAN DER MARK, P., AYRES, D. L., DARLING, A., HOHNA, S., LARGET, B., LIU, L., SUCHARD, M. A. & HUELSENBECK, J. P. 2012. MrBayes 3.2: efficient Bayesian phylogenetic inference and model choice across a large model space. *Systematic biology,* 61**,** 539-42.

SCHUBART, C. D., CUESTA, J. A. & FELDER, D. L. 2002. Glyptograpsidae, a new brachyuran family from Central America: Larval and adult morphology, and a molecular phylogeny of the Grapsoidea. *Journal of Crustacean Biology,* 22**,** 28–44.

SIMON, C., BUCKLEY, T. R., FRATI, F., STEWART, J. B. & BECKENBACH, A. T. 2006. Incorporating Molecular Evolution into Phylogenetic Analysis, and a New Compilation of Conserved Polymerase Chain Reaction Primers for Animal Mitochondrial DNA. *Annual Review of Ecology, Evolution, and Systematics,* 37**,** 545-579.

TSANG, L. M., CHAN, T. Y., AHYONG, S. T. & CHU, K. H. 2011. Hermit to king, or hermit to all: multiple transitions to crab-like forms from hermit crab ancestors. *Systematic biology,* 60**,** 616-29.

TSANG, L. M., MA, K. Y., AHYONG, S. T., CHAN, T. Y. & CHU, K. H. 2008. Phylogeny of Decapoda using two nuclear protein-coding genes: origin and evolution of the Reptantia. *Molecular phylogenetics and evolution,* 48**,** 359-68.

WIENS, J. J. 2003. Missing Data, Incomplete Taxa, and Phylogenetic Accuracy. *Systematic biology,* 52**,** 528-538.

WIENS, J. J. & TUI, J. 2012. Highly incomplete taxa can rescue phylogenetic analyses from the negative impacts of limited taxon sampling. *PLoS One,* 7.
